# Glycerol Monolaurate and Dodecylglycerol Effects on *Staphylococcus aureus* and Toxic Shock Syndrome Toxin-1 In Vitro and In Vivo

**DOI:** 10.1371/journal.pone.0007499

**Published:** 2009-10-19

**Authors:** Ying-Chi Lin, Patrick M. Schlievert, Michele J. Anderson, Christina L. Fair, Matthew M. Schaefers, Ramaiah Muthyala, Marnie L. Peterson

**Affiliations:** 1 Department of Experimental and Clinical Pharmacology, College of Pharmacy, University of Minnesota, Minneapolis, Minnesota, United States of America; 2 Department of Microbiology, Medical School, University of Minnesota, Minneapolis, Minnesota, United States of America; 3 Center for Orphan Drug Research, University of Minnesota, Minneapolis, Minnesota, United States of America; University of Liverpool, United Kingdom

## Abstract

**Background:**

Glycerol monolaurate (GML), a 12 carbon fatty acid monoester, inhibits *Staphylococcus aureus* growth and exotoxin production, but is degraded by *S. aureus* lipase. Therefore, dodecylglycerol (DDG), a 12 carbon fatty acid monoether, was compared in vitro and in vivo to GML for its effects on *S. aureus* growth, exotoxin production, and stability.

**Methodology/Principal Findings:**

Antimicrobial effects of GML and DDG (0 to 500 µg/ml) on 54 clinical isolates of *S. aureus*, including pulsed-field gel electrophoresis (PFGE) types USA200, USA300, and USA400, were determined in vitro. A rabbit Wiffle ball infection model assessed GML and DDG (1 mg/ml instilled into the Wiffle ball every other day) effects on *S. aureus* (MN8) growth (inoculum 3×10^8^ CFU/ml), toxic shock syndrome toxin-1 (TSST-1) production, tumor necrosis factor-α (TNF-α) concentrations and mortality over 7 days. DDG (50 and 100 µg/ml) inhibited *S. aureus* growth in vitro more effectively than GML (p<0.01) and was stable to lipase degradation. Unlike GML, DDG inhibition of TSST-1 was dependent on *S. aureus* growth. GML-treated (4 of 5; 80%) and DDG-treated rabbits (2 of 5; 40%) survived after 7 days. Control rabbits (5 of 5; 100%) succumbed by day 4. GML suppressed TNF-α at the infection site on day 7; however, DDG did not (<10 ng/ml versus 80 ng/ml, respectively).

**Conclusions/Significance:**

These data suggest that DDG was stable to *S. aureus* lipase and inhibited *S. aureus* growth at lower concentrations than GML in vitro. However, in vivo GML was more effective than DDG by reducing mortality, and suppressing TNF-α, *S. aureus* growth and exotoxin production, which may reduce toxic shock syndrome. GML is proposed as a more effective anti-staphylococcal topical anti-infective candidate than DDG, despite its potential degradation by *S. aureus* lipase.

## Introduction

*Staphylococcus aureus* is an important cause of skin and mucosal infections both in hospital and community settings [1], [2]. Approximately 20% of the U.S. population is persistently colonized by *S. aureus* in the nose, and 30% are intermittently colonized [1]. People colonized with *S. aureus* are at higher risk of becoming infected by the organism, especially when host defenses are breached, for example, postsurgical wounds, catheter insertions, or burn wounds. People suffering from atopic dermatitis are more likely to be colonized and infected by *S. aureus* than the general population. Superantigens produced by *S. aureus* are known factors that enhance skin inflammation in atopic dermatitis and may be responsible for steroid resistant T cell responses [3].

Superantigens, especially toxic shock syndrome toxin 1 (TSST-1), are also responsible for systemic exotoxemias such as toxic shock syndrome (TSS), an acute onset and potentially life-threatening illness. Clinical manifestations of TSS include fever, hypotension, rash, desquamation, and multi-organ failure. These symptoms are the result of overwhelming cytokine production systemically due to abnormal cross-linkage between T cells and macrophages by superantigens. The most recognized cases of TSS are associated with tampon usage in menstruating women; however, TSS is also associated with *S. aureus* infections at surgical or skin infection sites [2], [4]. Bacterial contamination of wound dressings, in particular occlusive dressings, have been suggested as the source of infection in some TSS cases [5].

Based on pulsed-field gel electrophoresis (PFGE), *S. aureus* strains can be grouped into several clonal types. In the United States, USA200 PFGE type were the most common methicillin susceptible *S. aureus* (MSSA) isolates recovered from national nasal colonization studies [6]. USA200 clonal type, which is genetically similar to the epidemic hospital strain EMRSA16 in the United Kingdom, is also the major clonal type associated with TSS, presumably due to the high prevalence of these isolates to possess *tst* (the gene for TSST-1). USA300 and USA400 clonal types have been associated recently with necrotizing pneumonia and necrotizing fasciitis in community settings [7]. Methicillin resistant *S. aureus* (MRSA) USA300 has emerged as one of the major causes of invasive staphylococcal infections in both community and hospital settings [7], [8].

Protein-synthesis inhibitors, such as clindamycin and linezolid inhibit TSST-1 production at sub-growth inhibitory concentrations, and the suppression is associated with improved clinical responses in patients with TSS [9]–[11]. However, these antibiotics are often recommended for treating antibiotic resistant *S. aureus* infections, and not for TSS prophylaxis. On the other hand, β-lactam antibiotics induce or increase TSST-1 production, which may increase the risk of TSS in patients with severe staphylococcal infections, especially by MRSA [12]. Given that most *S. aureus* infections are initiated at mucosal and skin sites, topical anti-staphylococcal agents, that can be incorporated into wound dressings, disposable medical devices, or tampons to inhibit toxin production and/or *S. aureus* growth and thus prevent *S. aureus* infections or TSS, would have clinical value.

Glycerol monolaurate (GML) (2,3-dihydroxypropyl dodecanoate) is a lauric acid glycerol ester commonly used in the food and cosmetic industries as an emulsifier and preservative and is generally recognized as safe (GRAS) by the Food and Drug Administration for topical use at doses up to 100 mg/ml. GML interferes with membrane signal transduction and thereby inhibits the growth of *S. aureus*, blocks the induction of *β*-lactamase, and delays the production of *S. aureus* exoproteins, such as TSST-1 and *α*-toxin [13]–[15]. GML also reduces the production of proinflammatory cytokines and chemokines by mammalian cells in response to *S. aureus* and purified TSST-1 (100 µg/ml), and prevents lethality in rabbits challenged vaginally with TSST-1 [16], [17]. Given these properties, GML has been tested as a tampon additive and reduces staphylococcal exotoxin production in vivo [18]. In addition, GML (5% gel) has been demonstrated recently to prevent vaginal SIV transmission in monkeys by inhibiting innate inflammatory responses [19]. The compound, however, is not stable in the presence of *S. aureus* and can be hydrolyzed by *S. aureus* esterase (lipase) into glycerol and lauric acid [13], [15]. To overcome the limitation of inactivation, compounds with ether linkage have been suggested as potential alternatives to GML. Many of these ether compounds inhibit TSST-1 production in addition to *S. aureus* growth, and they are more stable than ester compounds (such as GML) to chemical and enzymatic hydrolysis [20], [21]. 1-O-Dodecyl-rac-glycerol (DDG) (3-(dodecyloxy)propane-1,2-diol) is the corresponding alkylglycerol ether to GML. DDG inhibits the growth of *Enterococcus faecium* and *Streptococcus mutans* primarily by stimulating autolysin activity and interfering with cell wall synthesis [21]–[25]. DDG simultaneously inhibits *S. aureus* growth and TSST-1 production, but the mechanisms of action has not been characterized [20].

Given the structural similarity of GML and DDG and its supposed stability to lipase degradation over GML, DDG was hypothesized to be more potent than GML at inhibiting *S. aureus* growth and TSST-1 production, and therefore a better antistaphylococcal agent candidate than GML. Our goal was to compare the efficacy of these two compounds on *S. aureus* growth, and TSST-1 production in vitro and in vivo. We also studied in vivo the interactions between host innate immune responses and the compounds during *S. aureus* infection.

## Results

### Stability against *S. aureus* enzymes

**Figure 1:**
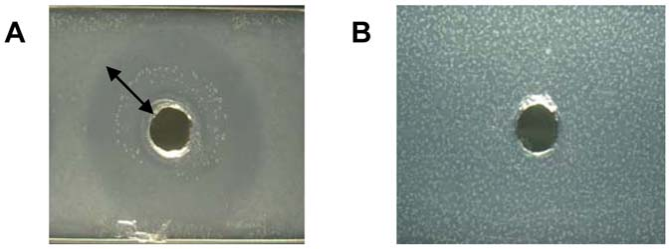
Stability of the compounds to *Staphylococcus aureus* (MN8) lipase. (A) Glycerol monolaurate (GML). (B) Dodecylglycerol (DDG). Clear zone indicates that the compound was degraded. Arrow denotes of the radius of the clear zone on the slide.

GML- and DDG-containing agarose slides were exposed to *S. aureus* overnight cultures to determine the stability of the compounds to lipase contained in the culture supernates. A clear zone was observed on the GML 500 µg/ml agarose slide, but not the DDG 500 µg/ml slide (Fig. 1). The solubility limit of GML in aqueous solutions at 37°C is approximately 100 µg/ml, and thus the zone of clearance, reflecting GML degradation by lipase, can be observed in the presence of a turbid background. The observation indicated that DDG was resistant to degradation by *S. aureus* MN8 lipase, while GML was not resistant to lipase.

### In vitro growth inhibition

**Figure 2:**
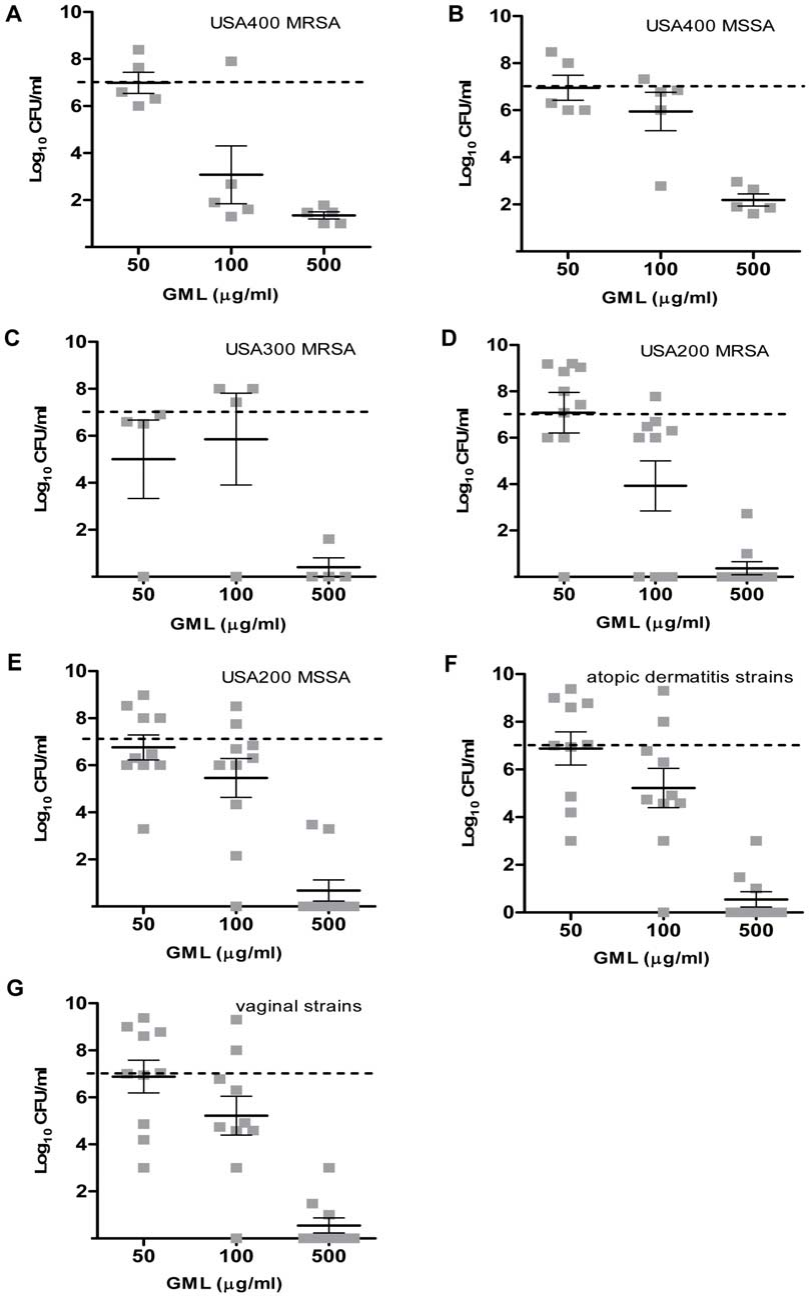
Glycerol monolaurate (GML) inhibition of *Staphylococcus aureus*. GML concentrations 50, 100, and 500 µg/ml were tested versus *S. aureus* isolates from different PFGE types, USA400 MRSA (A), USA400 MSSA (B), USA300 MRSA (C), USA200 MRSA (D), USA200 MSSA (E), atopic dermatitis strains (F), vaginal strains from healthy women (G), for 18 h at 37°C with shaking. The dashed line indicates the starting inocula. Each square (▪) indicates one isolate. The bars represent the mean±SEM of bacterial density in the group.

**Figure 3:**
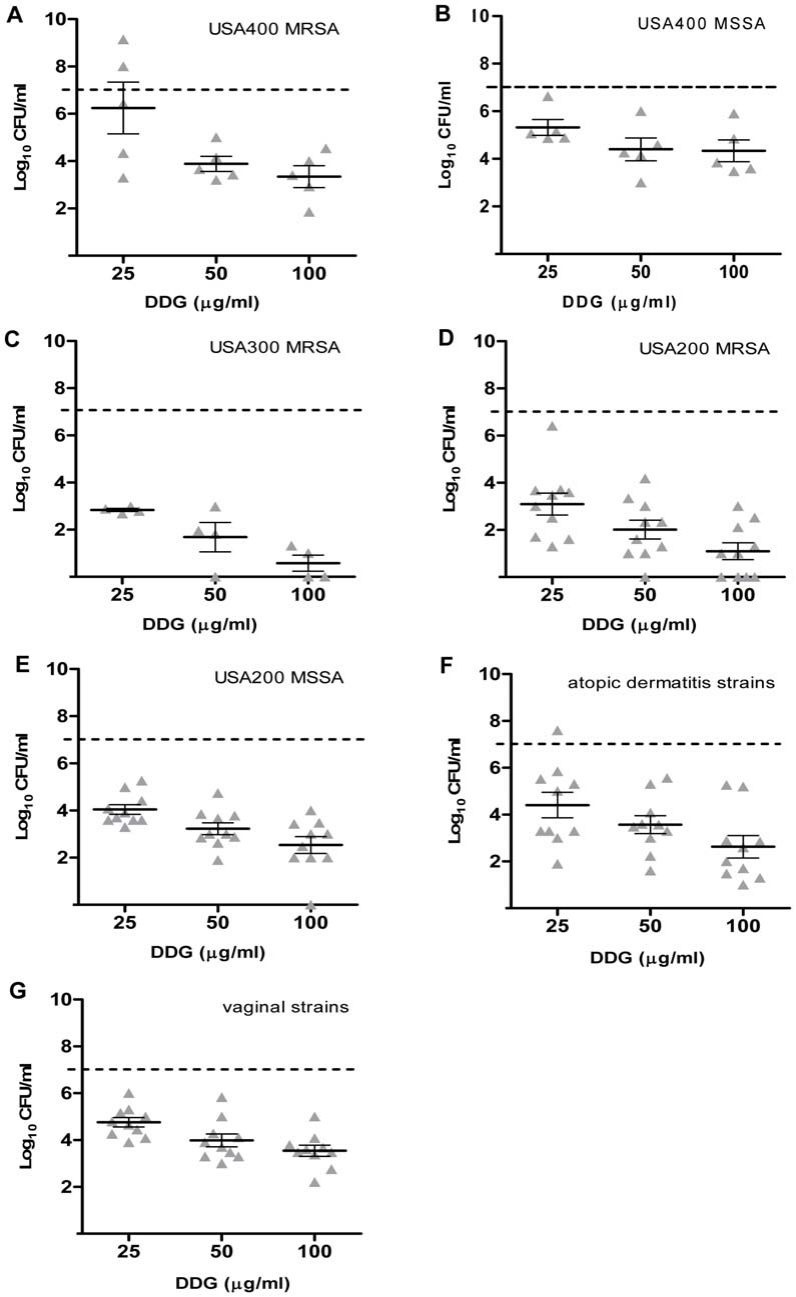
Dodecylglycerol (DDG) inhibition of *Staphylococcus aureus*. DDG concentrations 25, 50, and 100 µg/ml were tested versus *S. aureus* isolates from different PFGE types, USA400 MRSA (A), USA400 MSSA (B), USA300 MRSA (C), USA200 MRSA (D), USA200 MSSA (E), atopic dermatitis strains (F), vaginal strains from healthy women (G), for 18 h at 37°C with shaking. The dashed line indicates the starting inocula. Each triangle (▴) indicates one isolate. The bars represent the mean±SEM of bacterial density in the group.

The differences in susceptibility to GML and DDG among *S. aureus* strains were evaluated broadly using a large collection of clinically relevant isolates (MSSA USA200, MRSA USA200, MRSA USA300, MSSA USA400, MRSA USA400, vaginal isolates from healthy women, and isolates from persons with atopic dermatitis). Growth inhibitory effects of GML (50, 100, and 500 µg/ml) and DDG (25, 50, and 100 µg/ml) were examined at 18 h. In general, GML was bacteriostatic at concentrations of 50 µg/ml and 100 µg/ml, and was bactericidal (3 log decrease in CFU/ml from the starting inoculum of 1×10^7^ CFU/ml) at the concentration of 500 µg/ml (Fig. 2). On the other hand, DDG had a bacteriostatic effect on most strain categories at the concentration of 25 µg/ml (one dilution lower than GML). As the concentrations of DDG increased from 25 to 50 and 100 µg/ml, bacterial densities decreased an additional 1–2 log CFU/ml (Fig. 3). Overall, DDG was consistently more effective in preventing bacteria growth among all *S. aureus* strain categories, including vaginal and atopic dermatitis strains, than GML at concentrations of 50 and 100 µg/ml (p<0.01 for comparisons of GML and DDG against all 54 strains at both concentrations). There was no significant difference between MSSA and MRSA in response to GML (p = 0.79 and 0.12 for GML 50 and 100 µg/ml, respectively); However, MRSA apear to be more susceptible to DDG than MSSA at the concentrations 50 and 100 µg/ml (p = 0.01 and p<0.01, respectively). Some clonal variability was noted among strains, where USA400 (MSSA and MRSA) strains were relatively more resistant to DDG and the high dose of GML (500 µg/ml) than other clonal types (USA300 and USA200) tested. Community-associated MRSA USA300 strains were the most susceptible clonal type to DDG.

### In vitro TSST-1 suppression

**Figure 4:**
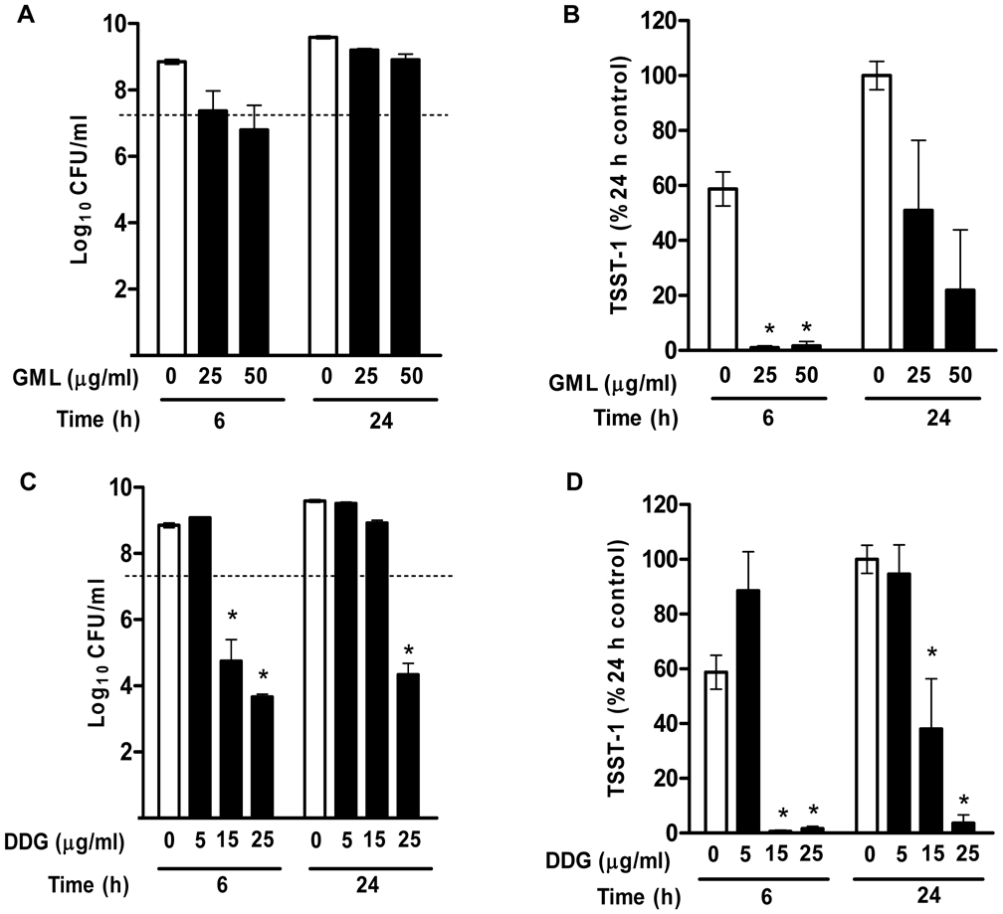
Effects of GML and DDG on *Staphylococcus aureus* Toxic Shock Syndrome Toxin-1 (TSST-1) production. (A) *S. aureus* MN8 was exposed to GML 0, 25 and 50 µg/ml for 6 and 24 h, and bacterial densities at 6 and 24 h were determined by plate counts. (B) The corresponding concentrations of TSST-1 of the above GML experiment. (C) *S. aureus* MN8 was exposed to DDG 0, 5, 15, and 25 µg/ml for 6 and 24 h. (D) The corresponding concentrations of TSST-1 from above DDG experiments. TSST-1 concentrations are presented as percent of the TSST-1 concentrations in 24 h control samples. Results are mean±SEM. The dashed line indicates the starting inocula. *, p<0.05.

To evaluate toxin inhibitory effects of the compounds at sub-growth-inhibition concentrations, *S. aureus* MN8 was tested for growth inhibition and the corresponding TSST-1 production by GML (25 and 50 µg/ml) and DDG (5, 15, and 25 µg/ml) at 6 and 24 h. GML (25 and 50 µg/ml) inhibited bacterial growth at 6 h, however, this bacteriostatic effect was no longer seen by 24 h (Fig. 4A). TSST-1 level was significantly reduced by GML at 25 and 50 µg/ml at 6 h (>99% reduction), and this effect persisted through 24 h (49% and 78% reduction, respectively) (Fig. 4B). DDG (5 µg/ml) did not inhibit the growth of MN8, and did not inhibit TSST-1 production (Fig. 4C). However, DDG (15 µg/ml) inhibited the growth of MN8 at 6 h but not 24 h, and TSST-1 production was inhibited by >99% at 6 h and 61% at 24 h (Fig. 4D). The results indicated that GML delayed TSST-1 production independent of growth inhibition, while DDG inhibition of toxin production was dependent on bacterial growth inhibition.

### Mammalian cell toxicity

**Figure 5:**
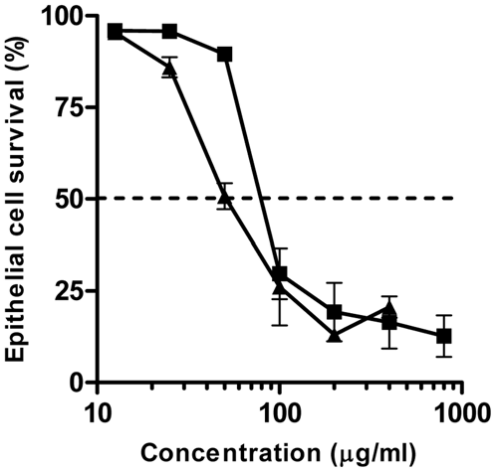
Cytotoxicity of GML and DDG to Human Vaginal Epithelial Cells (HVECs). HVECs were exposed to GML (▪) and DDG (▴) for 6 h. Cytotoxicity was accessed by measuring the release of LDH. Error bars are SEM. The dashed line indicates median cell survival (LD_50_). Symbols:▪, GML; ▴, DDG.

Since GML and DDG are most likely to be utilized in topical applications, they will be in contact with epithelial cells. Therefore, we determined the toxicity (median lethal dose, LD_50_) of GML and DDG to immortalized human vaginal epithelial cells (HVECs) using an assay to measure the membrane integrity (lactate dehydrogenase [LDH] release) following 6 h incubations. The LD_50_ of GML for a monolayer of confluent HVECs was 83 µg/ml (95% confidence interval [CI]: 69–99 µg/ml), while the LD_50_ of DDG for HVECs was 50 µg/ml (95% CI: 43–62 µg/ml) (Fig. 5). These results indicated that DDG was statistically more toxic to HVECs than GML. However, since the LD_50_ concentrations of DDG were lower than its bacterial growth inhibition concentrations in vitro, the compound may still be useful as an antistaphylococcal agent.

### In vivo rabbit Wiffle ball infection model

**Figure 6:**
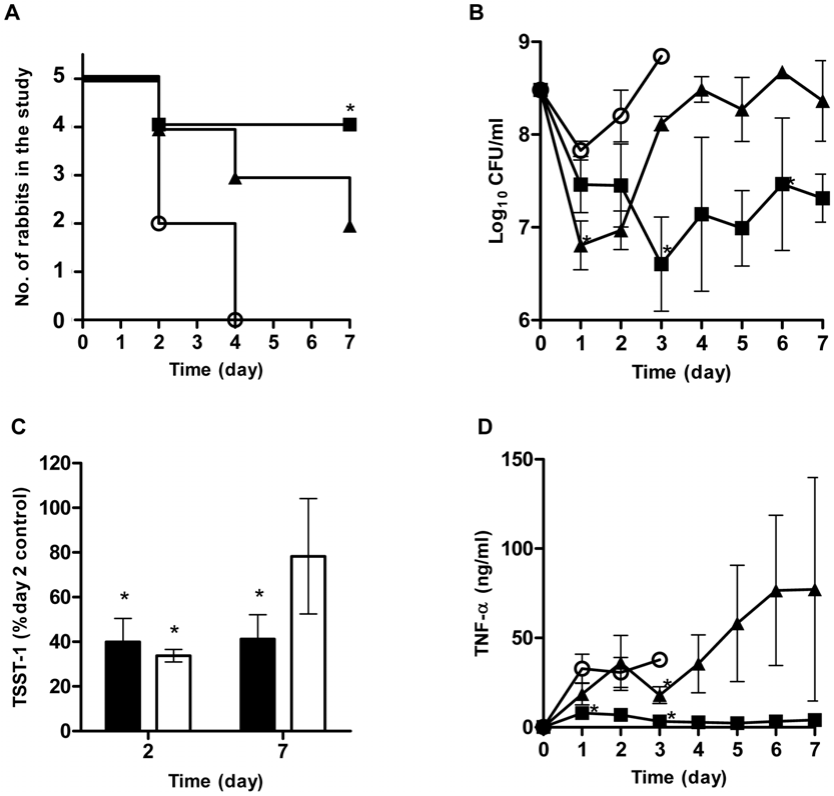
Antistaphylococcal effects of GML and DDG in a rabbit Wiffle ball infection model. Rabbits (n = 5 in each group) were infected with 3×10^8^ CFU/ml *S. aureus* MN8, and compounds (final concentration 1 mg/ml) were instilled into the Wiffle balls every-other-day and rabbits monitored up to 7 days. Survival of the rabbits (A), bacterial counts (B), TSST-1 production (C), and TNF-α levels (D) in the Wiffle balls. TSST-1 presented as percent of day 2 TSST-1 concentrations of the control rabbits (GML, close bars; DDG, open bars). Error bars are SEM. Symbols: ○, control; ▪, GML; ▴, DDG; *, p<0.05.

Both GML and DDG demonstrated in vitro potential as topical anti-staphylococcal agents, thus their efficacy in vivo was evaluated using a rabbit Wiffle ball infection model with compound (1 mg/ml, every-other-day) injected directly into the site of infection [see Materials and Methods]. This model is a model for toxic shock syndrome (TSS) as the bacteria are localized in the Wiffle ball both in suspension and as biofilms formed along the Wiffle ball surface; however, superantigens penetrate the Wiffle ball encapsulation tissue into blood circulation to cause systemic effects, including TSS [26], [27]. The survival curves for these experiments are shown in Fig. 6A. All rabbits in the control group (N = 5) died by day 4 (3 on day 2 and 2 on day 4) following inoculation of the Wiffle balls with *S. aureus* (MN8) 3×10^8^ CFU/ml. Two of 5 rabbits in DDG group and 4 of 5 rabbits in GML group were alive by the end of the 7 day study. One rabbit in DDG group died on day 2, 1 on day 4, and 1 on day 7; one rabbit in GML group died on day 2. The survival of rabbits receiving GML (4/5) was statistically better than the control (0/5) group (p<0.05, Fisher exact).

The Wiffle ball infection model provides the opportunity to study the interactions among bacteria, host innate immune responses, and therapeutic compounds by taking repeated samples at the infection site over time. We had difficulty obtaining fluids from Wiffle balls in two rabbits (one of the rabbits in GML and one in DDG group).Thus, the sample analyses, including bacterial counts, TNF-α (as a biomarker for TSS), and TSST-1 levels, were based on available sample points. All Wiffle ball sample fluids uniformly contained *S. aureus*, but *Pasteurella multocida*, was recovered from a sample taken on day 2 of a rabbit in the DDG group. That rabbit died at day 4.

One day after initial dosing, bacterial counts in the DDG treatment group were 0.65 and 1 log lower than those in the GML treatment and the control groups, respectively (Fig. 6B). However, in spite of the repeated dosing, bacterial counts in DDG group increased over time. In contrast, GML suppressed bacterial growth throughout the 7 day study period. Neither compound was able to achieve >3 log reduction in bacterial load within the Wiffle ball at the concentration tested.

TSST-1 concentrations within the Wiffle ball cavities of GML and DDG treated rabbits were lower than the control rabbits (Fig. 6C). On day 2, TSST-1 was significantly inhibited by 60% and 66% of the control rabbits for GML and DDG, respectively (p<0.001, both treatments). On day 7, TSST-1 in the GML group remained significantly lower than TSST-1 in control rabbits on day 2 (58% reduction, p<0.05), while the level in DDG group was not significantly lower than controls on day 2 (22% reduction).

Baseline TNF-α levels in the Wiffle ball fluids of all rabbits were below the lower limit of detection (200 pg/ml) prior to infection (Fig. 6D). One day after bacterial challenge, rabbits treated with GML showed significantly lower concentrations of TNF-α in the Wiffle ball cavities than rabbits in control and DDG treatment groups: TNF-α average concentrations increased to 32.8 ng/ml (range: 11.6–61.9 ng/ml), 7.9 ng/ml (range: 1.8–17.7 ng/ml), and 18.6 ng/ml (range: 3.1–95.9 ng/ml) for control, GML, and DDG groups, respectively. TNF-α concentrations of rabbits in the GML group remained low throughout the period of study (4.1 ng/ml; range: 3.0–5.5 ng/ml). On the other hand, there was wide variation in TNF-α in the DDG group. TNF-α in one rabbit decreased from 26.9 ng/ml on day 1 to 14.7 ng/ml on day 7, but other rabbits in this group had increasing TNF-α levels throughout the experiment. Overall, there is no strong evidence indicating DDG's ability to modulate innate immune responses.

## Discussion

TSS is a serious complication of *S. aureus* infection, and the superantigen, TSST-1, is responsible for nearly all menstrual TSS cases and at least half of non-menstrual cases [28]. Many surfactants, including fatty acids linked through ester, ether, amide, or amine bonds, appear to inhibit *S. aureus* growth and toxin production [20]. However, fatty acid esters and amides are susceptible to *S. aureus* enzyme degradation, and amines are irritable to mucous membranes [29]. Therefore, fatty acid ethers were considered to be better candidates as topical anti-staphylococcal agents. Lauric acid (a 12 carbon-containing fatty acid) was determined to be the most potent saturated fatty acid when C8 to C18 -containing fatty acids were tested against gram-positive bacteria [30]. Its ester derivative, GML, has shown excellent potential for being incorporated into tampons to reduce risk of TSS [18]. However, similar to other fatty acid esters, GML is susceptible to *S. aureus* lipase degradation [13]. DDG, the corresponding ether to GML, was therefore compared to GML as an anti-staphylococcal candidate to reduce the risk of TSS. The studies presented in this manuscript determined that GML and DDG inhibit *S. aureus* growth and toxin production, although by apparent different modes of action. In addition, a difference in strain (clonal type) specific susceptibility to both GML and DDG was observed. Overall, the studies indicated that GML is a potentially better anti-staphylococcal agent than DDG for its ability to inhibit exoprotein production regardless of effects on bacterial growth, to reduce mortality in the rabbit Wiffle ball infection model, and to cause less cytotoxicity to epithelial cells than DDG.

A range of minimum inhibitory concentrations (MICs) for GML against *S. aureus* have been reported. The MICs of GML against 29 strains of *S. aureus* in a complex medium were reported to be between 10 to 20 µg/ml with 10^3^ to 10^4^ CFU/ml inocula [31]. Kabara and colleagues reported the MIC of GML against *S. aureus* was 25 µg/ml with approximate 10^7^ CFU/ml inocula in trypticase soy broth [30]. Preuss *et al.* reported 63 µg/ml with approximate 10^5^ to 10^6^ CFU/mL inocula in nutrient broth [32]. Kelsey and colleagues reported the MIC of GML against three strains of *S. aureus* was 25–50 µg/ml [33]. The variability in MIC is potentially due to culture conditions, inoculum size, and the *S. aureus* strains tested [31]. By testing the compounds against a large collection of clinical relevant strains, we confirmed that there are differences in sensitivity to GML among bacterial strains, even within the same clonal type. The differences in GML sensitivity may not be solely explained by different levels of lipase produced by the strains since the differences among *S. aureus* clonal types can also be observed in the DDG group, which is not degraded by *S. aureus* lipase. We also observed that USA400 strains do not produce more lipase than USA200 strains (data not shown), despite being more resistant to the compounds. The mechanism(s) behind the differences among *S. aureus* clonal types in response to DDG and GML may be related to cell surface hydrophobicity [34], however, this hypothesis will need to be investigated in future studies.

Although structurally similar, DDG and GML interact with *S. aureus* differently. Glycerol esters are commonly found in bacterial membranes, and cells have mechanisms to maintain membrane integrity in their presence. This is likely to occur also in the presence of GML. In contrast, glycerol monoethers are uncommon in bacterial membranes, and thus, the bacteria may be expected to have greater difficulty in maintenance of membrane integrity in the presence of DDG. As noted in our studies, GML antimicrobial effects were dose dependent and required higher concentrations for bactericidal activity, while DDG was predominantly bacteriostatic, but active at lower concentrations than GML. Similar mechanisms may also explain the differences between GML and DDG on toxin inhibition. GML blocks toxin induction by interfering with bacterial signal transduction on bacterial cell membranes [14]. In our study, GML (25–50 µg/ml) was able to inhibit TSST-1 production independent of *S. aureus* growth inhibition properties, which is in agreement with previously described results by Schlievert et al. (20 µg/ml) [15], and Holland et al. (17 µg/ml) [31]. DDG was also reported to inhibit TSST-1 production by McNamara and colleagues [20]. However, our results suggest that DDG, and likely other glycerol monoethers, inhibition of toxin production is dependent on bacterial growth inhibition, which is different from that of GML.

GML has a good safety profile on skin and mucosal surfaces. The compound was considered to have negative ocular irritation and have a LD_50_ of >20 g/kg for rats when dosed orally for 10 weeks [35]. In fact, GML (5% gel) was safe for chronic vaginal administration in monkeys over a 6 month test period [17]. In unpublished studies with year-long passage of *S. aureus* MN8 on sub-growth-inhibitory concentrations of GML, we observed no increase in resistance to GML's antimicrobial and anti-exotoxin effects. On the other hand, the safety of DDG in vivo has not been well studied. In one study, mice given 1 g/kg of DDG orally per day over 4 weeks indicated no signs of toxicity, and DDG was quickly absorbed and eliminated into urine [36]. Since increased doses of DDG do not enhance bacterial growth inhibition in our study, and DDG may be more irritable to mucosal surfaces than GML, minimal effective dose of DDG should be used.

GML has been reported to stabilize the membrane of eukaryotic cells, modulate the production of pro-inflammatory cytokines and thereby prevent the toxicity of bacterial exotoxins on eukaryotic cells [16]. We have previously suggested the benefits of GML as a dual-acting anti-infective, 1) with effects on the microbes to prevent growth and/or exotoxin production, and 2) with anti-inflammatory and membrane stabilizing effects on the host epithelial cells, which reduces the disruption in the mucosal permeability barriers caused by induction of pro-inflammatory cytokines and chemokines following infection [19]. This latter anti-inflammatory and membrane stabilizing property, although counterintuitive, may be equally important or more important than the antimicrobial effect. We reported recently that a GML (5%) containing gel prevented SIV transmission across monkey cervical and vaginal mucosa, despite mucosal surface GML concentrations being below virucidal concentrations [19]. Additionally, histological studies demonstrated an inhibitory effect on innate immunity. In our study, GML also decreased local pro-inflammatory cytokine production (as measured by TNF-α) despite bacterial densities of approximately 1×10^7^ CFU/ml over 7 days.

Production of TSST-1 is induced by elevated oxygen and carbon dioxide levels, neutral pH, presence of proteins, and 37°C [37]. Introduction of oxygen into the typically anaerobic vaginal environment may account for the tampon association with TSS [38], [39]. Although abscesses are typically perceived to be anaerobic, Todd and colleagues demonstrated that *S. aureus* abscesses are aerobic, and appear to provide TSST-1 stimulating environmental conditions, similar to those occurring vaginally in the presence of tampons [40]. The Wiffle ball infection model as used in this study has aspects of both types of *S. aureus* infections included in an aerobic encapsulated abscess, which has internal surfaces similar to the vaginal mucosa. As bacteria were encapsulated in the Wiffle ball, the model provided an ideal environment for real-time monitoring of the interactions among the host innate immune response, bacteria, and treatments (DDG and GML) at the infection site.

Based on the collective results of this study, GML is proposed as more effective anti-staphylococcal topical anti-infective candidate than DDG, despite its potential degradation by *S. aureus* lipase.

## Materials and Methods

### *S. aureus* isolates

Fifty-four clinical isolates were tested to assess the ability of GML versus DDG to inhibit the growth of *S. aureus*. These included 10 menstrual vaginal TSST-1^+^ MSSA isolates within the pulsed-field gel electrophoresis (PFGE) type USA200 as defined by the CDC [41]. These isolates were from TSS patients across the United States. Ten TSST-1^+^ MRSA isolates were included within PFGE type USA200, and all of these isolates were from Minnesota, with 6 from patients with TSS. Five isolates were USA400 MRSA, and 5 isolates were USA400 MSSA. All USA400 isolates were from patients with necrotizing pneumonia, purpura fulminans, or non-menstrual TSS [42]. Three of the USA400 MRSA and three of the USA400-related MSSA isolates made the superantigen staphylococcal enterotoxin C (SEC), and two in each group made SEB. All ten isolates were positive for Panton-Valentine leukocidin (PVL). Four isolates were categorized as USA300 MRSA and were positive for the superantigen enterotoxin-like Q and made PVL [41]. Since GML and DDG are likely to be used topically, *S. aureus* derived from both skin and mucous membranes were also evaluated. Vaginal isolates (N = 10) were obtained from healthy women during menstruation, and 10 skin strains were obtained from patients with atopic dermatitis. These 20 isolates were not further characterized with respect to exotoxin production or methicillin susceptibility. Collectively, these 54 clinical isolates were isolated from 1995 to 2007 and are maintained in the Schlievert and Peterson laboratories in the lyophilized state as low passage cultures.

*S. aureus* MN8 is a USA200 MSSA clinical isolate whose growth and exotoxin responses to GML have been reported previously [14], [15]. Therefore, this strain was chosen to evaluate exotoxin inhibitory ability of the compounds and used in the rabbit Wiffle ball infection study.

### Antimicrobial compounds

DDG (racemic 1-*O*-dodecylglycerol; CAS registry number: 1561-07-5; 3-(dodecyloxy)propane-1,2-diol; Alexis Corporation, Läufeltinger, Switzerland), and glycerol monolaurate (GML) (Monomuls 90-L12; CAS registry number: 142-18-7; 2,3-dihydroxypropyl dodecanoate; Cognis, Cincinnati, Ohio) were prepared as high concentration stocks. GML was dissolved in ethanol, and DDG was dissolved in dimethyl sulfoxide (DMSO), as recommended by the manufacturers.

### Determination of compound degradation by bacterial lipase

Overnight *S. aureus* (MN8) culture supernates (20 µl) were filtered to remove bacteria and placed into wells on agarose slides incorporated with either GML or DDG (500 µg/ml) and incubated for 5 h at 37°C and degradation of compound was assessed visually by measuring zone of clearing. The method for preparing these slides was adapted from Schlievert et al. [15].

### Culture conditions

Bacteria were cultured overnight in Todd-Hewitt (TH) Bacto broth (Becton Dickinson and Company, Sparks, MD) at 37°C with 200 revolutions per minute (RPM) shaking. Experiments were performed with approximate starting inoculum of 1×10^7^ colony-forming units (CFU)/ml with various concentrations of GML and DDG in 1 ml of TH broth. Samples (50 µl) were serially diluted with phosphate buffered saline (PBS; Cellgro-Mediatech Inc., Herndon, VA), and spirally plated onto sheep blood agar (Becton Dickinson). Plates were incubated at 37°C overnight and CFU counted using aCOLyte Supercount computer software (Microbiology International, Frederick, Maryland). The lower limit of accuracy was 400 CFU/ml, approximately 2.6 log_10_ CFU/ml. For *S. aureus* MN8 experiments (6 h and 24 h), an additional 300 µl of samples were collected and frozen for TSST-1 quantification. Bactericidal activity was defined as a 99.9% (3-log10 reduction in CFU/ml) reduction in bacterial density at 18–24 h compared to the initial inoculum. The term bacteriostatic was used when bacterial growth compared to the initial inoculum was either not observed or reduced by less than 99.9% [43].

### TSST-1 Western blotting

Proteins in the 300 µl bacterial culture supernates were concentrated by precipitation with 4 volumes of 100% ethanol and re-suspended in 60 µl of sterile distilled water. Rabbit Wiffle ball supernate samples were not concentrated. For Western blotting, samples [1∶1 mixed with Laemmli sample buffer (Bio-Rad Laboratory, Hercules, CA)] were separated by sodium dodecyl sulfate polyacrylamide gel electrophoresis (SDS-PAGE, 12% acrylamide) [44]. After transfer to polyvinylidene fluoride membranes (Bio-Rad), the membranes were sequentially incubated with primary anti-TSST-1 (Toxin Technology. Inc., Sarasota, FL), secondary anti-rabbit IgG-alkaline phosphatase (Sigma-Aldrich), and 5-bromo 4-chloro 3-indolyl phosphate/nitroblue tetrazolium (Sigma-Aldrich) for development [45]. The relative band density was determined with ImageJ (version 1.40 g; http://rsb.info.nih.gov/ij/).

### Cytotoxicity of the compounds to human vaginal cells

Immortalized human vaginal epithelial cells (ATCC CRL-2616) were used to determine mammalian cell cytotoxicity of GML and DDG. The cells were maintained in Keratinocyte-Serum Free medium (KSFM, GIBCO-BRL, Grand Island, NY), supplemented with recommended supplements and antibiotics/antifungal (100 IU/ml penicillin, 100 µg/l streptomycin, and 2.5 µg/ml Fungizone). Cells were seeded into 96-well plates and grown to confluency. Cells were changed to antibiotic/antifungal-free KSFM the day before experimentation. Cells were co-incubated with compounds for 6 h at 37°C in a humidified incubator with 7% CO_2_. CytoTox-One homogenous membrane integrity assay (Promega) was used to measure the release of lactate dehydrogenase (LDH) from damaged cells as an indicator of membrane integrity. Assays were performed according to the manufacturer's instructions. Absorbances at 560 nm (excitation) and 590 nm (emission) wavelengths were measured by SpectraMax M2 microplate reader (Molecular Devices, Sunnyvale, CA). All experiments were performed in triplicate. Median lethal doses (LD_50_) of the compounds were the intercepts of 50% cell survival and the regression line of the two points adjacent to the values.

### Rabbit Wiffle ball infection model

Ethics statement: All animal experiments were performed in accordance with protocols approved by the University of Minnesota Institutional Animal Care and Use Committee (IACUC). The rabbit Wiffle ball infection model has been previously described [27], [46]. Briefly, golf-ball-sized Wiffle balls were implanted subcutaneously in the flanks of Dutch-belted rabbits (either sex, 1.5 to 2.5 kg). The animals were allowed to recover for 6–8 weeks. On day 0 of experimentation, the animals (n = 5) received 0.3 ml of 100 mg/ml GML or DDG (or solvent control) by injection directly into the Wiffle ball (final concentration of 1 mg/ml in the 30 ml Wiffle ball). A 1 mg/ml (0.1%) final concentration for DDG and GML was chosen and hypothesized to be non-toxic and efficacious as we previously determined that a GML (5%) containing gel inserted vaginally every day in monkeys for 6 months was not toxic [17]; and initial in vitro results indicated this concentration of GML and DDG would be bactericidal against *S. aureus* and inhibit TSST-1 production. The same treatments were administered every other day (days 2, 4, and 6). Overnight *S. aureus* MN8 cultures were grown in TH broth, washed once with PBS, and re-suspended to the desired concentration in PBS. Immediately after injecting the compounds, 1×10^10^ CFU *S. aureus* MN8 (in a volume of 1.0 ml) was injected into each animal's Wiffle ball (30 ml), bringing the local concentration to approximately 3×10^8^ CFU/ml. Animals were monitored daily for signs of TSS, including fever (with use of rectal thermometers), diarrhea, weight loss, and moribundity (as an indication of imminent death). A small volume of fluid (0.3 ml) was drawn from each Wiffle ball daily for bacterial counts, TSST-1 measurement, and TNF-α determination. Animals were euthanized on day 7.

### Tumor necrosis factor α (TNF-α) ELISA

TNF-α was used as a biomarker of inflammation at the infection site. Purified recombinant rabbit TNF-α, capture antibody, primary detection antibody (goat anti-rabbit TNF-α), secondary anti-rabbit antibody (biotin mouse anti-rabbit TNF-α), and assay reagents were commercially available from Becton Dickinson. Rabbit Wiffle ball fluids were diluted a minimum of 1∶2 with assay buffer to eliminate viscosity and nonspecific effects. Lower limit of detection of this assay was approximately 200 pg/ml.

### Statistical methods

Paired *t*-tests were performed to compare the differences in bacterial densities between GML and DDG (50 and 100 µg/ml) against the 54 clinical *S. aureus* isolates. Un-paired *t*-tests were used to compare the susceptibility of MRSA and MSSA. Total *S. aureus* CFU/ml and TSST-1 levels among groups were compared using one-way analysis of variance (ANOVA) with *Bonferroni* method to adjust *p* values for multiple comparisons. Fisher's exact test was used to compare rabbit survival between treatment groups. A *p*≤0.05 was considered statistically significant. Computations and graphing were performed using Prism version 5 (GraphPad Software, Inc. La Jolla, CA).
